# Exploring the Role of P2X Receptors in Alzheimer’s Disease

**DOI:** 10.3389/fphar.2019.01330

**Published:** 2019-11-07

**Authors:** Pamela Andrea Godoy, Oscar Ramírez-Molina, Jorge Fuentealba

**Affiliations:** Neuroactive Compounds Screening Laboratory,Departamento de Fisiología, Facultad de Cs. Biológicas, Universidad de Concepción, Concepción, Chile

**Keywords:** purinergic receptors, P2X receptors, amyloid beta, Alzheimer’s disease, adenosine triphosphate

## Abstract

Several studies have pointed to soluble oligomers of beta amyloid peptide (SOAβ) as the principal neurotoxic agents responsible for the generation of synaptotoxic events that can explain the main symptoms of Alzheimer’s disease (AD). Among the toxic features associated with SOAβ, one of the most notorious is the formation of a non-selective pore-like structure in the plasma membrane, which may partly explain the overload of intracellular Ca^2+^. There is evidence that the pore causes leakage of key intracellular compounds, such as adenosine triphosphate (ATP), to the extracellular milieu. Extracellular ATP activates P2X receptors (P2XR), which are ligand-gated ion channels (LGICs) widely expressed in both neuron and glial cells and act as neuromodulators of synaptic activity by promoting Ca^2+^ entry and facilitating neurotransmitter release. There is abundant evidence correlating the overexpression of these receptors to neurodegenerative diseases, including AD, thus opening the possibility that P2XR could potentiate the toxic mechanisms induced by SOAβ and contribute to intracellular Ca^2+^ overload in neurons and other mechanisms related to glial activation and inflammation. In this review, we correlate scientific evidence related to the main toxic effects induced by SOAβ and those that are mediated by purinergic P2XR. The data suggest that these purinergic receptors participate in the deleterious cellular and molecular effects of SOAβ that lead to the pathogenesis of AD. This information sheds light on the participation of new components in SOAβ toxicity that could be interesting as pharmacological targets for the development of molecular or chemical compounds able to modulate them.

## Introduction

### Alzheimer’s Disease and Aβ Peptide

Alzheimer’s disease (AD) is a neurodegenerative disorder characterized by the progressive loss of memory and decline in other cognitive skills. AD is the main cause of dementia that affect the elderly and it is expected that by the year 2050 152 million people around the world will be affected (Philip Scheltens et al., 2016; Patterson, 2018). Sadly, there are no effective treatments despite efforts to find different therapeutic approaches (Patterson, 2018). There are two classical histopathological hallmarks in AD: amyloid plaques and neurofibrillary tangles (Ballard et al., 2011). Amyloid plaques are composed of insoluble aggregates of Aβ peptide, while neurofibrillary tangles are formed by hyperphosphorylated tau protein (Kocahan and Doğan, 2017). The pathogenesis of AD has not been completely elucidated yet, but one of the theories that tries to explain the origin of this disease is the amyloid hypothesis (Hardy and Higgins, 1992; Selkoe, 2000; Karran et al., 2011), a theory that has changed and evolved over time. Today, it is proposed that increased levels and aggregation of Aβ are important to the development of AD (Ballard et al., 2011), and that soluble oligomers of Aβ are the pathogenic form of this peptide (Lal et al., 2007; Cline et al., 2018).

### Amyloid-β Peptide: Pore Formation and Leakage of Adenosine Triphosphate (ATP)

It is now largely accepted that the beta-amyloid peptide (Aβ) is the main toxic agent in Alzheimer’s disease. Aβ aggregation generates small soluble oligomers, larger insoluble oligomers, fibrils, and finally, one of the hallmarks of this disorder, the senile plaques (Mudher and Lovestone, 2002). Aβ peptide is generated by the cleavage of the Amyloid Precursor Protein (APP), a transmembrane protein that can be proteolytically processed through two pathways: the non-amyloidogenic that involves the participation of the α and γ secretases, and the amyloidogenic in which the β and γ secretases produce the Aβ peptide (Thinakaran and Koo, 2008). Different studies suggest that the soluble oligomers (SOAβ) are the main form of aggregated Aβ that generates synaptotoxic events (Lal et al., 2007; Shankar et al., 2007; Krafft and Klein, 2010). Several toxic events induced by SOAβ have been described such as acute cytosolic Ca^2+^ increase (in a concentration-dependent manner), reduced synaptic vesicle recycling and depletion of these vesicles (Parodi et al., 2010), impairment in mitochondrial dynamics and mitochondrial dysfunction (Wang et al., 2007), increment in oxidative stress, and inflammation (Varadarajan et al., 2000; Ballard et al., 2011). Furthermore, a direct interaction of the Aβ peptide with some membrane receptors such as α7 nicotinic receptor and the cellular prion protein has been described (Wang et al., 2000; Peters et al., 2015). Also, the excitotoxicity effect of Aβ peptide with NMDA receptors has been widely studied and correlated (Zhang et al., 2016). This peptide impairs LTP through NMDA receptors, and this impairment can be ameliorated by decreasing glutamate levels (Zhang et al., 2016). Under chronic conditions, the Aβ peptide decreases NMDA levels and its presence in the membrane thereby reducing glutamatergic transmission (Zhang et al., 2016). On the other hand, memantine, an NMDA antagonist, is used to treat some stages of AD because it helps to modulate the high calcium levels that are potentiated by NMDA receptors (Wang and Reddy, 2017). It seems that Aβ peptide impairs glutamate uptake allowing its increased availability in the extracellular milieu (Wang and Reddy, 2017), and this can contribute to the excitotoxicity observed in acute stages of AD, and chronic depletion that is characteristic in advanced stages (Sáez-Orellana et al., 2016). Another remarkable toxic effect that has been described is the capacity of the Aβ peptide to generate pore-like structures in the plasma membrane (Arispe et al., 1993; Mobley et al., 2004; Parodi et al., 2010). Although the mechanism for this pore formation is still under discussion, several studies suggest that the pore allows the passage of different molecules through a non-selective channel, and that soluble oligomers are the species responsible for its formation (Lal et al., 2007; Fuentealba et al., 2011; Sáez-Orellana et al., 2016; Kandel et al., 2017; Lee et al., 2017). This pore formation in the plasma membrane can explain some of the classic toxic events seen after treatment with SOAβ, such as the non-controlled increase in intracellular calcium [(Ca^2+^)_i_] levels that generate an overload of this cation in the cytosol (Mattson et al., 1992; Berridge, 2010; Parodi et al., 2010; Sepulveda et al., 2010), which could lead to the observed mitochondrial dysfunction and neuronal death (Kruman and Mattson, 1999). It has been proposed that the pore generated by the Aβ peptide starts as a cationic selective channel and later transitions to a non-selective pore that allows the passage of molecules up to 900Da of weight such as EtBr and 6-NBDG, a glucose fluorescent analog (Sepúlveda et al., 2014). Besides the entry of some molecules into the cell, this pore can produce a leakage of some relevant intracellular compounds such as ATP. Indeed, several studies showed an increment in the extracellular levels of this molecule induced by Aβ peptide treatment (Kim et al., 2007; Fuentealba et al., 2011; Sáez-Orellana et al., 2016). The extracellular increase in ATP (by leakage through the pore or some other mechanism such as co-secretion of ATP with other neurotransmitter) could be associated with a reactive glia response. ATP can generate calcium transients in glial cells, and it appears to contribute to astrocytic hyperactivity in a mouse AD model (Delekate et al., 2014). Therefore, some authors have proposed that ATP participates in the inflammation process widely described in this neurodegenerative disease (Heppner et al., 2015; McGeer et al., 2016), while others propose that ATP, through some P2X receptors (P2XR), could participate directly in some of the toxic SOAβ effects observed in neurons (Sáez-Orellana et al., 2016; Sáez-Orellana et al., 2018).

### ATP and Purinergic Receptors

Purinergic receptors are activated by purine molecules, and the two main families are P2Y and P2X receptors. P2Y are G protein-coupled receptors composed of 8 different subunits, whereas P2XR are from the ligand gated ion channel (LGIC) family and 7 different subunits have been described in mammals (Burnstock, 2006; Kaczmarek-Hájek et al., 2012; Khakh and North, 2012). When P2XR are activated by ATP, they allow the passage of cations and have a high Ca^2+^ permeability (Khakh and North, 2012). To see a more extensive review on P2XR and their role in neurological disorders see Sáez-Orellana et al. (2015). ATP has been described to play an important role in inflammation because it is present in the extracellular environment in inflammatory processes where purinergic receptors can be activated (Molz et al., 2015). ATP can be released through traumatic processes such as necrosis and also by controlled release through other mechanisms like hemichannels (Idzko et al., 2014) and co-secretion with neurotransmitters like catecholamine (Gandía et al., 1987; Xu et al., 1991; Estévez-Herrera et al., 2016). Once in the extracellular milieu, ATP can activate P2 receptors and subsequent activation of several different signaling cascades. The upregulation of some P2XR, such as P2X7, have been reported on immune cells, where several evidence demonstrate their involvement in different inflammation processes (Burnstock, 2016). In this cell type ATP triggers the release of inflammatory mediators as cytokines and prostaglandin E2 (PGE2) (Burnstock, 2016). P2X7 appears to be the most important P2XR on inflammation, this receptor is expressed on immune cells such as mast cells, neutrophils, macrophages and microglia, where it has a proinflammatory role, by causing the inflammasome assembly and secretion of IL1β (Idzko et al., 2014; Karmakar et al., 2016). In this review, we discuss in more depth the role of some ionotropic purinergic receptors (P2XR) in the Aβ peptide related toxicity and Alzheimer’s disease, a neurodegenerative disorder where inflammation is a central mechanism.

## Alzheimer’s Disease and Purinergic Ionotropic Receptors

### P2X7 and Inflammation

The P2X7 coding sequence was obtained from brain and peripheral ganglion, and later it was described that this purinergic receptor is expressed in microglia and ependymal cells from the central nervous system (CNS) (Collo et al., 1997). High concentrations of ATP (in the low millimolar range) are needed to activate P2X7R (EC_50_ 100 µM for the rat receptor) (Khakh and North, 2012; Di Virgilio et al., 2018). It has been widely described that the P2X7 purinergic receptor participates in different inflammatory processes (Di Virgilio et al., 2017), and therefore, could play a key role in chronic inflammation and neuropathic pain. Indeed, a study done in P2X7 knockout (KO) mice found that these animals did not present inflammation or neuropathic pain (Chessell et al., 2005). This purinergic receptor has been found in all immune cells and is upregulated in inflammatory processes; and it has also been related to most neurodegenerative diseases (Di Virgilio et al., 2018). Therefore, the most studied P2XR in the context of AD is P2X7, and some light has been shed on its possible participation in the pathogenesis mechanism of this neurodegenerative disorder. A study using Tg2567 transgenic mice that overexpress APP with the Swedish mutation found an overexpression of P2X7 in 24 month old mice when compared to age match controls, with most of the staining surrounding amyloid plaques (Parvathenani et al., 2003). Furthermore, it has been described the overexpression of P2X7 in microglia and astrocytes from patients with AD (McLarnon et al., 2006; Martin et al., 2019). It has also been found that this receptor was overexpressed in cultured fetal human microglia exposed to Aβ, and that the cells treated with the peptide elicited a larger calcium influx by the agonist BzATP, which suggests a functional overexpression of this purinergic receptor. The same overexpression was observed in hippocampus from rats injected with Aβ, where the immunostaining was associated mainly with microglia (McLarnon et al., 2006). Results from another study showed that Aβ induced ROS production in a primary microglia culture that was mediated by the increment in intracellular calcium through P2X7R activation by extracellular ATP (Kim et al., 2007). Other studies demonstrated that microglia cells lacking P2X7R did not show an increment in intracellular calcium and ATP release after exposure to Aβ, but wild type cells expressing P2X7 did (Sanz et al., 2009). Furthermore, the use of oATP (a P2XR blocker) in wild type cells also inhibited the ATP release after Aβ exposure (Sanz et al., 2009). Finally, it was found that Aβ induced the release of IL-1β from a primary microglia culture and this effect was blocked by apyrase, while microglia from a KO mouse for P2X7 did not release IL-1β after Aβ exposure (Sanz et al., 2009). Also, intrahippocampal injection of Aβ in mice produced an accumulation of IL-1β, but in P2X7 KO mice the release of this cytokine was significantly lower (Sanz et al., 2009). Microglia not only release inflammatory cytokines, they also phagocytize the Aβ peptide. It has been described that silencing of P2X7 or the use of Brilliant Blue G (BBG), a non-competitive antagonist of P2X7 with a nanomolar affinity, and over 1,000-fold more potent than at P2X4 (Jiang et al., 2000), on microglia cells not only inhibited the release of pro-inflammatory cytokines such as IL-1β and TNF-α, but also increased Aβ phagocytosis by these cells (Ni et al., 2013). The results from this work suggested that the increment in IL-1β mediated by the activation of P2X7 produced an impairment in Aβ phagocytosis. Moreover, the administration of BBG to mice injected intrahippocampally with Aβ showed an improvement in cognitive and spatial learning function (Fan et al., 2014). This same study observed that BBG prevented the reduction in dendritic filopodia and density of spines induced by Aβ in hippocampal primary cultures (Fan et al., 2014). Recently, a more in-depth study of P2X7 expression was performed in a transgenic mouse model produced by breeding the J20AD mouse model (which overexpresses APP with the Swedish and Indiana mutations) and ^P2X7R^EGFP reporter mice, which expresses this fluorescent protein under the control of the P2X7 promoter (Martinez-Frailes et al., 2019). In this model, they observed that in advanced stages of the disease, once microgliosis could be detected, the expression of P2X7 increased in microglial cells, and the number of microglial cells that expressed P2X7 also increased and were mainly localized on senile plaques. The data suggested that this effect was caused by the neuroinflammation produced in these mice, and that ATP could act as a chemotaxis signal for microglial cells through the activation of P2X7, which lead the microglial cells to the senile plaques. Interestingly, they also found that P2X7R activation reduced the phagocytic capacity of these cells (Martinez-Frailes et al., 2019). Recently, it has been described that in the APPPS1 transgenic mice P2X7R deficiency reduces the cognitive impairment and the number of Aβ plaques and soluble species, although they did not observed significantly differences in the Aβ phagocytosis and the activation state of microglia (Martin et al., 2019). Despite the fact that they did not observe changes in the expression of cytokines, such as IL-1β and TNFα, they demonstrated that APPPS1 mice deficient for P2X7 present lower cerebral levels of chemokines (CCL3, CCL4, and CCL5) than in the APPPS1 transgenic mice (Martin et al., 2019), together with this they observed a reduced CD8^+^ T cell recruitment in the choroid plexus and hippocampus. Therefore, P2X7 appears to be involved in the chemokine production and T cell recruitment induced by Aβ in the APPPS1 transgenic mice (Martin et al., 2019).

The inflammatory process is an important feature in the pathology of AD; therefore some reports have suggested a protective role for nonsteroidal anti-inflammatory drugs (NSAIDs) (Vlad et al., 2008; Zurita et al., 2013). All of these results suggest an important role for ATP and P2X7 in the inflammatory response of the microglia cells in AD through the release of cytokines such as IL-1β and the impairment of Aβ phagocytosis.

### P2X4 in Inflammation and Neuronal Death

The P2X4 receptor was cloned from rat brain where it was found to be expressed in neurons and blood vessels (Soto et al., 1996). The gene that codes for P2X4 in humans is located on chromosome 12, close to the P2X7 gene, and they were likely produced by local gene duplication (Suurväli et al., 2017). It was found that P2X4 increased its expression in P2X7 KO mice suggesting that these receptors could complement their function (Suurväli et al., 2017). The crystal structure of P2X4 from zebrafish was the first P2X receptor to be observed revealing that these are trimeric receptors (Kawate et al., 2009). P2X4 has also been correlated with some inflammation processes. For instance, P2X4R appears to participate in joint inflammation and osteoarthritis, where it induces the expression of IL-1β, TNFα and participates in the inflammasome formation (Fan et al., 2014; Li et al., 2014). Also, it has been described that in human monocyte-derived macrophages P2X4R activated by ATP and through Ca^2+^ influx modulates the expression and secretion of the chemokine CXCL5, therefore, this receptor could participate in the neutrophils recruitment (Layhadi et al., 2018). In the CNS, it has been demonstrated that P2X4 increased its expression in microglial cells after exposure to LPS, which enhanced the sensitivity and the response to low ATP concentrations (Raouf et al., 2007). Furthermore, an upregulation of P2X4 in activated microglial cells after brain ischemic injury was also observed and was related to the increase in extracellular ATP levels (Cheng et al., 2014). Surprisingly, it was found that P2X4 protein levels were decreased in the middle frontal gyrus and middle temporal gyrus from AD patients with severe cognitive impairment (Varma et al., 2009). On the other hand, in hippocampal neurons exposed to the Aβ peptide, P2X4 levels were increased after 6 h of treatment, and this receptor expression was observed in cell bodies; while after 12 h of exposure to Aβ, P2X4 was observed in cell bodies and neurites (Varma et al., 2009). Also, an accumulation of a smaller P2X4 fragment was found after exposure to Aβ, and this was prevented by using a caspase inhibitor suggesting that this cleavage of P2X4 by caspase impaired the trafficking induced by ATP exposure (Varma et al., 2009). This cleavage could change some properties of these channels, generating a bigger amplitude response and a slower closure time in response to ATP (Varma et al., 2009). Finally, overexpression of P2X4 in neurons enhanced the toxic effect of Aβ, while silencing of this purinergic receptor decreased cellular death after exposure to Aβ indicating that this purinergic receptor contributed to the neuronal cell death induced by Aβ (Varma et al., 2009).

Hence, P2X4R could participate in some inflammatory processes in AD, together with P2X7R, since similarities between these two subtypes have been described; and potentiating the toxic effects of Aβ peptide and increasing neuronal death. Additionally, it seems interesting the recently advances discussed in the literature (Stokes et al., 2017), about the inhibition of these receptors. It has been described that some antidepressants such as paroxetine, inhibits P2X4 (Abdelrahman et al., 2017), but this molecule is not selective for P2X4, being more potent inhibiting the reuptake of serotonin (Stokes et al., 2017). Recent research have led to the discovery of for more selective antagonist for this receptor, like the benzodiazepine derivative 5-BDBD and compounds derived from N-substituted phenoxazine (Stokes et al., 2017). This opens a new space for the pharmacological developing of new drugs capable to modulate the P2X4R.

### P2X2 Upregulation

The P2X2 receptor was first described in rat pheochromocytoma PC12 cells (Brake et al., 1994). Later, it was demonstrated to have a wide expression in the central nervous system. For example, its immunoreactivity was dense in some important areas such as the olfactory bulb, cerebral cortex, amygdala and hippocampus (Kanjhan et al., 1999). Hippocampal pyramidal cells from CA1 to CA4 and granular cells of the dentate gyrus express high levels of P2X2 and the immunostaining is mostly confined to the soma and dendrites (Kanjhan et al., 1999). In general, a strong P2X2 expression was associated to neurons in different CNS areas. Its presence in dendritic spines supports the idea that ATP can act as a fast excitatory neurotransmitter, whereas the presynaptic localization is consistent with the report of purinergic receptors acting as neurotransmitter release modulators (Kanjhan et al., 1999). The expression of heteromeric P2X2/3 receptors in neurons from the dorsal root ganglion and their involvement in neuropathic pain has been described (Ueno et al., 2003). It was found that P2X2 and P2X3 mRNA increased in mouse models of neuropathic pain, and results suggested that this heteromeric receptor was involved in mechanical allodynia in this mouse model (Ueno et al., 2003). This upregulation could be in the presynapse of dorsal root ganglion neurons facilitating the mechanical stimuli transmission that could cause mechanical allodynia (Ueno et al., 2003). The upregulation of P2X2 (along with P2X4) has also been described in an *in vitro* ischemic model (Cavaliere et al., 2003). The addition of suramin (a P2 antagonist) during the ischemic insult prevented cell death, and moreover, the silencing of P2X2 decreased the ischemic damage (Cavaliere et al., 2003). The same increase in P2X2 was also observed in the CA1–CA2 pyramidal cell layer, strata oriens and radiatum using immunostaining in an *in vivo* animal model of ischemia (Cavaliere et al., 2003). P2X2 colocalized with neuronal markers, but not with glial markers, confirming the neuronal expression of P2X2, while P2X4 was mainly observed in glial cells (Cavaliere et al., 2003). The P2X2 overexpression in neurons and the prevention of cellular damage by silencing P2X2 suggest a role for this purinergic receptor in neuronal degeneration. Our group and others have described that after exposure to Aβ there was a consistent increment in intracellular calcium, but we found that co-treatment with apyrase or PPADS significantly reduced this increment (Sáez-Orellana et al., 2016). The same could be observed for the increment in frequency and amplitude of excitatory activity by acute exposure to Aβ (Sáez-Orellana et al., 2016). Therefore, this data supports the idea that Aβ induces excitotoxic events and P2XR would be contributing to this effect by facilitating the increment in intracellular calcium and synaptic facilitation (Sáez-Orellana et al., 2016). Chronic exposure to Aβ decreased SV2 and PSD95 proteins and this effect was prevented by PPADS, a P2XR antagonist. Furthermore, PPADS was able to prevent the decrease in cell viability observed with Aβ (Sáez-Orellana et al., 2018). Using qRT-PCR, a significant increase in mRNA for P2X1, 2, 5, and 7 was found after 12 h of exposure to Aβ, but after 24 h only P2X2 remained increased (Sáez-Orellana et al., 2018). The levels of P2X2 were analyzed with immunocytochemistry and western blot and the results also demonstrated an increment in P2X2 after 24 h of exposure to Aβ (Sáez-Orellana et al., 2018). Taken together, these results suggest that excitotoxic effects induced by Aβ are associated to changes in the purinergic tone, where an increase in extracellular ATP by leakage through the amyloid pore could cause an overexpression of purinergic receptors, as observed for P2X2, potentiating the acute and chronic toxic effects of Aβ.

## Discussion

Extracellular ATP and P2XR play an important role in several inflammatory processes, where the upregulation of some subunits have been described in different immune cells and they mediate the secretion of some inflammatory mediators. Therefore, ATP and ionotropic purinergic P2X receptors could play an important role in the pathogenesis of Alzheimer’s disease, a neurodegenerative disorder that exhibits a sustained inflammatory response.

**Figure 1:**
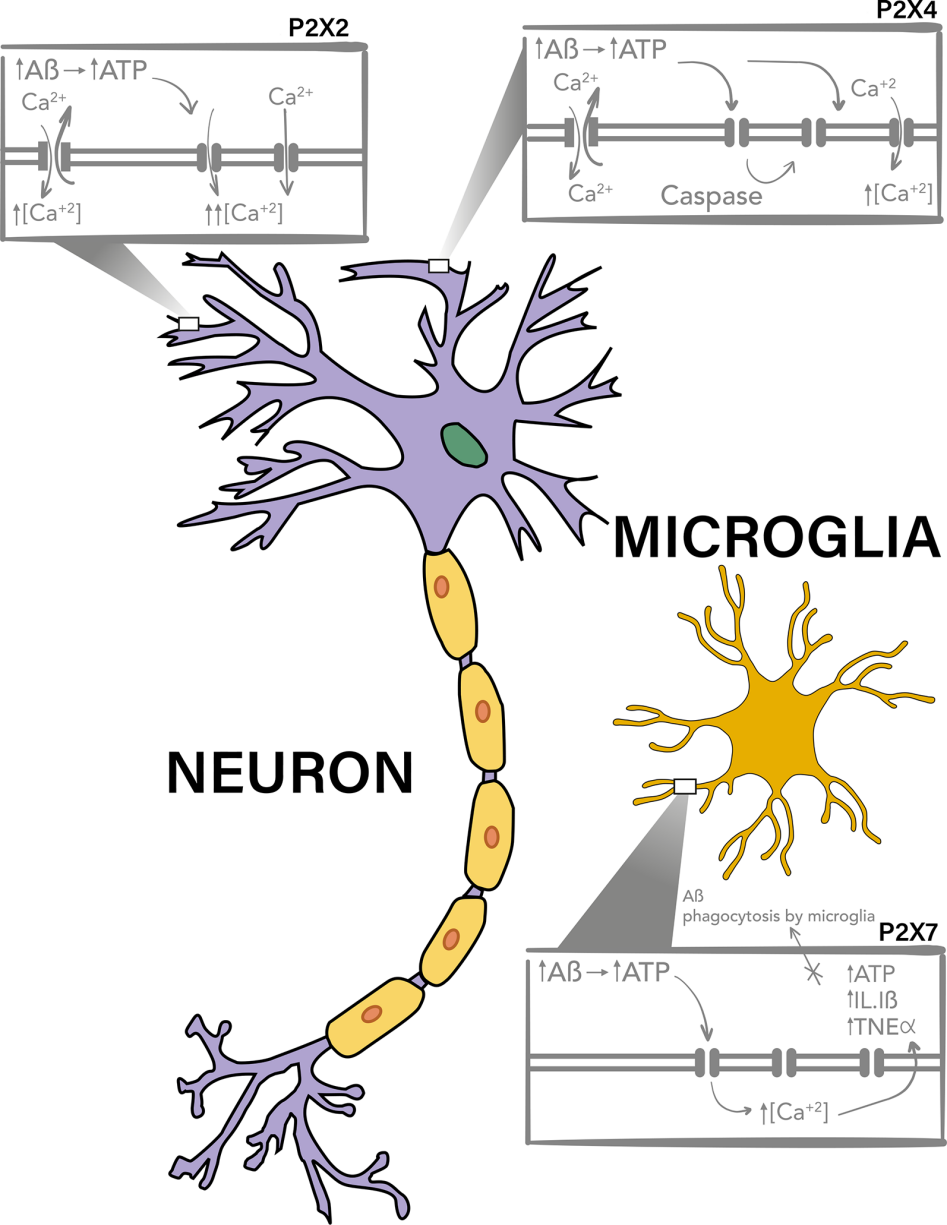
P2X7, P2X4, and P2X2 purinergic receptors are involved in Aβ toxicity. P2X4 and P2X2 have a neuronal expression and have been described to be upregulated and to participate in the toxic mechanisms of Aβ peptide, probably due to the potentiation of the increment on intracellular calcium levels after activation by ATP, which is increased in the extracellular milieu. It has been described that after Aβ treatments there is a P2X4 fragment produced by caspase which is accumulated in the plasma membrane and have different channel properties, allowing the activation of this receptor for longer periods of time. On the other hand P2X7 has a mainly microglial expression, and it has also been reported to overexpresses on Aβ toxicity models and AD. This purinergic receptor, also through increase in intracellular calcium levels, potentiate the release of pro-inflammatory cytokines and participates in the impairment of Aβ phagocytosis by the microglia.

P2X7 is the P2XR most studied on the pathogenesis of AD. This purinergic receptor has a prominent expression in glial cells and it appears to participate in the inflammatory response of microglia through the release of cytokines, chemokines and the impairment of Aβ phagocytosis (**Figure 1**, Lower box).

P2X4 is expressed in both microglia and neurons, and therefore it could participate in some inflammatory processes in AD, (**Figure 1**, upper right box), together with P2X7R, and in neuronal death.

Finally, some evidence show that purinergic receptors participate in the excitotoxic effects induced by Aβ, where their upregulation, as that observed for P2X2, could potentiate the toxic effects of the Aβ peptide (**Figure 1**, upper left box).

In conclusion, all the literature discussed in this review points to the P2XR family as potential therapeutic targets in the development of new treatments for Alzheimer’s disease.

## Author Contributions

PG and OR-M made the full literature recompilation and initial revision. PG and JF discussed the literature and wrote the text and OR-M designed the initial scheme.

## Funding

This work was funded by FONDECYT 1161078 (JF). PG is a PhD student of CONICYT (21160392).

## Conflict of Interest

The authors declare that the research was conducted in the absence of any commercial or financial relationships that could be construed as a potential conflict of interest.

## Abbreviations

AD, Alzheimer’s disease; APP, Amyloid Precursor Protein; ATP, Adenosine triphosphate; BBG, Brilliant Blue G; BzATP, 2,3′-O-(4-Benzoylbenzoyl) ATP; [Ca^2+^]i, Intracellular calcium; CNS, Central nervous system; EC_50_, Half maximal effective concentration; EGFP, Enhanced green fluorescent protein; EtBr, Ethidium bromide; KO, Knockout; LGIC, Ligand gated ion channel; LPS, Lipopolysaccharides; mRNA, Messenger RNA; NMDA, N-methyl-D-aspartate; NSAIDs, Nonsteroidal anti-inflammatory drugs; oATP, Oxidized ATP; P2XR, Ionotropic purinergic receptors; P2X2R, Ionotropic purinergic P2X2 receptor; P2X4R, Ionotropic purinergic P2X4 receptor; P2X7R, Ionotropic purinergic P2X7 receptor; P2Y, Purinergic G protein-coupled receptors; PC12, Rat adrenal gland pheochromocytoma; PGE2, Prostaglandin E2; PPADS, Pyridoxal phosphate-6-azo(benzene-2,4-disulfonic acid) tetrasodium salt Hydrate; PSD95, Post synaptic density protein 95; qRT-PCR, Real time reverse transcription polymerase chain reaction; ROS, Reactive oxygen species; SOAβ, Soluble oligomers of beta amyloid peptide; SV2, Synaptic vesicle protein 2; 6-NBDG, 6-(N-(7-Nitrobenz-2-oxa-1,3-diazol-4-yl)amino)-6-Deoxyglucose.
